# Teaching Academic Words With Digital Flashcards: Investigating the Effectiveness of Mobile-Assisted Vocabulary Learning for University Students

**DOI:** 10.3389/fpsyg.2022.893821

**Published:** 2022-06-14

**Authors:** Ismail Xodabande, Yasaman Iravi, Behzad Mansouri, Hoda Matinparsa

**Affiliations:** ^1^Department of Foreign Languages, Kharazmi University, Tehran, Iran; ^2^Department of Foreign Languages, Imam Khomeini International University, Qazvin, Iran; ^3^Research and Innovation Department, Lakeshore Foundation, Birmingham, AL, United States; ^4^Department of Foreign Languages, Islamic Azad University, Qazvin, Iran

**Keywords:** mobile-assisted vocabulary learning, academic vocabulary, digital flashcards, corpus-based language teaching, EAP

## Abstract

The current study explored the effects of using digital flashcards (DFs) and mobile devices on learning academic vocabulary. The participants were 86 university students majoring in Psychology in two experimental conditions and one control group. A list of 361 core academic words frequently used in Psychology was taught to the participants using different materials, and the learning outcomes were compared across the three groups. Accordingly, the participants in the experimental group 1 (*N* = 31) used a DF application (i.e., NAWL builder), participants in the experimental group 2 (*N* = 30) used traditional materials (i.e., paper flashcards), and those in the control group were given a list of target words with their definitions. Receptive knowledge of the target words was tested before and after the treatment, and the learning outcomes were compared across the groups using one-way between-groups ANOVA. The findings of the study indicated that using DFs enhanced students’ engagement with learning their discipline-specific academic vocabulary and that experimental group 1 outperformed those participants in other learning conditions. The findings add to the existing literature on mobile-assisted vocabulary learning and provide empirical support for the effectiveness of such platforms for learning academic vocabulary. The implications of the study were discussed in terms of the affordances provided by DFs on mobile devices and corpus-based word lists for informing vocabulary learning components in teaching English for Academic Purposes (EAP).

## Introduction

There is a consensus that learning a language is highly contingent upon mastery of its vocabulary (Nation, 2013; Webb and Nation, 2017). Vocabulary knowledge has been viewed as “the key type of knowledge necessary for any language use, because if words to express concepts are not known, all syntactic and discourse knowledge is of little use” (Schmitt et al., 2021, p. 10). The importance given to the development of vocabulary knowledge as an indicator of success or failure in any form of second or foreign language (L2) learning has contributed to pedagogical advancement specifically focused on enhancing the quality and quantity of words learned in an L2. For example, the development of vocabulary word lists such as General Service List (GSL; West, 1953), Academic Word List (Coxhead, 2000), and Knowledge-based Vocabulary Lists (Schmitt et al., 2021) all has been confirming the fact that knowing the words of a language and learning how to use them are probably the most essential aspect of the literacy in language learning in general and L2 learning in particular (Yang and Coxhead, 2020). Accordingly, finding effective strategies to facilitate the development of lexical competence among L2 learners remained a worthwhile research agenda.

Research pertinent to English vocabulary instruction has also shown that learning English words and developing L2 vocabulary knowledge either incidentally or intentionally could be a daunting task for learners situated in English as a foreign language (EFL) contexts (Laufer, 1996; Webb and Chang, 2012; Honzard and Soyoof, 2020). The whole process could also be accompanied by excessive pressure and extra load when it comes to learning words and vocabulary items not commonly found in everyday conversations (Coxhead, 2018, 2019; Yüksel et al., 2020). In other words, learning words known as technical and academic vocabulary could seem too overwhelming to learners that they may not actively participate in the process of learning. To ease some of the pressure exerted on both L2 teachers and learners, scholars have called for the inclusion and expansion of new technologies and findings of corpus linguistics research in L2 vocabulary teaching and learning (Ma, 2017; Coxhead, 2018, 2019; Ma and Mei, 2021; Soyoof et al., 2021). In this regard, the current study aimed to investigate the effectiveness of mobile devices and digital flashcard applications in learning academic words that are frequently used in the field of Psychology. The study contributes to the expanding body of knowledge on mobile-assisted vocabulary learning (Lin and Lin, 2019), and the findings might inform academic vocabulary instruction in English for Academic Purposes (EAP) programs (Soyoof et al., 2022).

## Literature Review

### Academic Vocabulary

Generally operationalized as the words used more frequently in academic texts and not in non-academic texts (Nation, 2013; Coxhead, 2019), academic vocabulary refers to a class of medium-frequency words (i.e., beyond high-frequency or general service vocabulary) that are used mainly for describing abstract ideas and processes in the scientific discourse and rhetorical organization of academic texts (Paquot, 2010; Coxhead, 2018). Recent corpus-based studies pointed to a considerably large coverage for academic vocabulary ranging from 6 to 14% in academic texts (Coxhead, 2000; Browne et al., 2013; Gardner and Davies, 2014), and it has been argued that knowledge of these words is crucial for understanding and producing academic writing and more generally academic literacy development (Coxhead and Byrd, 2007; Nagy and Townsend, 2012; Gardner and Davies, 2014). Moreover, research indicated that academic vocabulary poses major challenges in terms of the learning burden not only for English as second/foreign language (ESL/EFL) learners but also for native English-speaking students (Evans and Morrison, 2010, 2011; Spencer et al., 2017). Hence, given their importance and crucial role in academic discourse, a number of core academic word lists have been developed for setting vocabulary learning goals for university students (e.g., Coxhead, 2000; Browne et al., 2013; Gardner and Davies, 2014).

The Academic Word List (AWL; Coxhead, 2000) developed more than two decades ago, has remained a major resource for vocabulary instruction, materials development, and vocabulary assessment in EAP programs (Coxhead, 2011; McLean and Kramer, 2015). Nonetheless, a growing number of studies investigating the coverage of the AWL in different academic corpora started to challenge its position as the predominant source of core academic vocabulary relevant to a wide range of disciplines (Hyland and Tse, 2007; Gardner and Davies, 2014; Masrai and Milton, 2018). More specifically, the AWL has been criticized on various grounds including (1) the use of outdated GSL for defining general service vocabulary (Gardner and Davies, 2014), (2) containing a large number of general or only marginally academic words (Masrai and Milton, 2018), (3) the variation in the coverage of the list across disciplines (Liu and Han, 2015), and (4) using word families as the unit of counting vocabulary items which limits its pedagogical value (Gardner and Davies, 2014). In recent years, two core academic word lists, namely the New Academic Word List (NAWL; Browne et al., 2013), and the Academic Vocabulary List (AVL; Gardner and Davies, 2014), have been developed in response to the limitations associated with the AWL. These new lists showed considerable improvements in terms of their coverage in academic texts and also employed more pedagogically useful units (i.e., lemma and flemma; Brown et al., 2020) in operationalizing academic vocabulary. For example, Browne et al. (2013) developed the NAWL that contains 960 words based on a carefully selected academic corpus with 288 million words as part of the Cambridge English Corpus (CEC). General service and high-frequency vocabulary items accounted for 86% of the CEC, and the NAWL increased this coverage to around 92%. In this regard, learning the vocabulary items in the NAWL is of significant importance for university students and might be regarded as a more appropriate vocabulary learning goal. Mastery of the items in these lists facilitates achieving the minimum comprehension threshold for understanding academic discourse (Schmitt et al., 2011; Laufer, 2013).

### Incidental and Deliberate Vocabulary Learning

Developments in vocabulary knowledge in first language (L1) learning generally occur incidentally, which means that there is no conscious or explicit attention to learning words themselves, but the focus is on meaning in communicative interactions. In other words, incidental vocabulary learning has been regarded as a by-product of communication that is associated with some sort of meaningful input (Nation, 2013). A crucial factor in incidental vocabulary development then is the amount of input, as more input increases the chances of encountering new words and boosts the likelihood of picking the meaning from the context (Webb and Nation, 2017). However, in learning additional languages beyond the L1, creating the facilitative conditions for incidental vocabulary learning is not easily possible. More specifically, language learners in most EFL contexts have very limited exposure to the target language beyond the classroom which significantly impacts incidental vocabulary learning. Accordingly, there is a growing consensus that for L2 learners, deliberate and intentional learning accounts for most developments in vocabulary knowledge (Laufer, 2005). Research in this area clearly pointed to higher gains in intentional vocabulary learning compared to incidental learning conditions for L2 learners (Webb and Nation, 2017).

Intentional vocabulary learning for L2 learners might be undertaken in different ways. Besides using available resources such as dictionaries and course books in the classroom, a range of approaches including using flashcards, learning from word lists, writing tasks, serious games, and fill-in-the-blanks activities contribute significantly to vocabulary learning (Webb et al., 2020; Li and Hafner, 2022; Soyoof et al., 2022). In this regard, one of the most effective approaches for intentional learning, paper, and digital flashcards has been employed extensively to augment vocabulary learning among EFL learners (Nakata, 2019). In a recent study, Li and Hafner (2022) explored the impacts of using word cards on mobile devices to improve receptive and productive vocabulary knowledge among Chinese EFL learners. The findings indicated that although both digital and traditional flashcards contributed significantly to vocabulary knowledge development, digital flashcards produced better learning outcomes. Moreover, recent developments in digital technologies created appropriate conditions for facilitating intentional vocabulary development in the form of self-directed learning outside the classroom. For example, Xodabande et al. (2022) investigated self-directed and intentional vocabulary learning among Iranian EFL students with paper and digital flashcards, and their findings indicated that such strategies hold considerable potential to shortcut the long-term process of vocabulary learning.

### Mobile-Assisted Vocabulary Learning

Mobile-assisted language learning (MALL) has attracted considerable attention in recent years (e.g., Dashtestani, 2016; Hwang and Fu, 2019; Chen et al., 2020; Nazari and Xodabande, 2020, 2021; Rassaei, 2020, 2021; Burston and Giannakou, 2021; Dashtestani and Hojatpanah, 2021), and a large number of studies investigated the use of different delivery mediums and environments (such as SMS, MMS, and mobile applications) for learning ESL/EFL vocabulary (Mahdi, 2017; Lin and Lin, 2019). Overall, the findings from this growing body of knowledge indicated that the use of SMS/MMS and mobile applications including context-aware, gaming, and researcher-designed platforms contributed significantly to improvements in EFL learners’ vocabulary knowledge (Mahdi, 2017; Xodabande, 2017; Lin and Lin, 2019; Soyoof et al., 2021). Additionally, a growing number of studies are investigating the affordances of digital flashcards (DFs) in the form of mobile applications for vocabulary learning (e.g., Nakata, 2019; Seibert Hanson and Brown, 2020; Xodabande and Atai, 2020; Yüksel et al., 2020; Zhang et al., 2021; Xodabande et al., 2022). In this regard, although the number of studies focusing on teaching general vocabulary is increasing, research on using such platforms (e.g., Anki) for facilitating the development of academic or technical vocabulary knowledge remained limited (Honzard and Soyoof, 2020; Yüksel et al., 2020). As such applications provide opportunities for meaningful repetition of the target vocabulary items and scaffold the learning experience through a number of multimedia features, their integration into language teaching programs seems to be especially promising for teaching academic vocabulary (Mansouri and Mantero, 2019; Xodabande and Atai, 2020). Additionally, by making use of digital flashcards, language teachers can create targeted content and opportunities to “support learners’ self-directed study efforts and help them consolidate [their] vocabulary knowledge” (Yüksel et al., 2020, p. 2), thus elevating learners’ agency in keeping themselves accountable for the progress in learning.

Some studies investigated the use of mobile devices for learning academic and technical vocabulary among university students. In a quasi-experimental study, Yüksel et al. (2020) compared the effectiveness of DFs (i.e., Quizlet) and wordlists on 57 undergraduate pharmacy students’ technical word learning over 10 weeks by collecting data from the pre-treatment survey, two technical vocabulary tests, and the post-treatment survey. The results pointed to the high potential of DFs in technical vocabulary learning. Furthermore, learners provided an optimistic perspective on using DFs. In another study, Kohnke et al. (2019) developed an application (i.e., *Excel@EnglishPolyU)* and two vocabulary-based English language learning games for learning business vocabulary. The researchers then investigated the business vocabulary retention of 51 undergraduate students at a university in Hong Kong. Analysis of data revealed the positive impacts of mobile-gamified applications in vocabulary knowledge development. Similarly, Kohnke et al. (2020) explored the vocabulary retention of 159 ESL learners from four disciplines at Hong Kong University using an in-house mobile application specially designed to build a repertoire of field-specific academic words. Their findings from the analysis of pre- and post-tests including 120 vocabulary items revealed the beneficial impacts of mobile apps on field-specific word learning and retention. Honzard and Soyoof (2020) compared the effectiveness of using mobile apps and serious games on English word retention among 90 Iranian EFL learners. Placing participants in two groups, the researchers conducted pre-test, post-test, and delayed post-test and found that both approaches were influential in enhancing participants’ word retention with serious games having an edge over a mobile application. The authors argued for the inclusion of using games besides the conventional mobile applications commonly used for vocabulary learning and teaching.

With respect to learning academic vocabulary, Dizon (2016) probed the effectiveness of DF Quizlet in vocabulary development. The participants were nine EFL students in a Japanese university who studied Coxhead’s (2001) general academic vocabulary list (AWL) over 10 weeks, and the findings of pre- and post-tests indicated that students gained considerably from the DF application. Moreover, Xodabande and Atai (2020) studied the impacts of a mobile application on self-directed learning of academic vocabulary among 38 Iranian university students. The participants were divided into experimental and control groups, and the study adopted a pre-, post-, and delayed post-test design to investigate the effects of mobile-assisted vocabulary learning in the long run. The participants in the experimental group used a flashcard mobile application to learn vocabulary items from AWL (Coxhead, 2000), and those in the control group used traditional materials for learning the same vocabulary items. Although the findings of the study showed improvements for both experimental and control groups in terms of academic vocabulary knowledge, the impacts on the experimental group were significantly higher than that of the control group. The study highlighted the potential of mobile applications for learning academic vocabulary. Similar findings were also reported by Ashcroft et al. (2018), as they compared the effect of DFs and paper flashcards on general academic vocabulary development at various English proficiency levels. Despite this emerging evidence on the benefits of using digital flashcards for learning academic and technical vocabulary, there are recent calls for more thorough and long-term intervention-based studies to comprehensively examine the impacts of utilizing such technologies on L2 vocabulary learning.

## The Preset Study

Given the importance of academic vocabulary for university students and the positive learning outcomes reported for mobile-assisted vocabulary learning, the current study aimed to explore the impacts of using DFs for teaching academic vocabulary to Psychology students. The study also compared the learning outcomes attained from using DFs to paper flashcards and word lists. The study is significant since it addresses a number of gaps in the literature. First, as needs analysis studies indicated, academic vocabulary knowledge featured high among the language learning needs of Psychology students (e.g., Atai and Hejazi, 2019). In this regard, finding effective strategies to facilitate academic vocabulary learning contributes to their academic literacy and professional identity development. Second, as highlighted above, studies investigating the impacts of mobile-assisted vocabulary learning on specialized vocabulary (i.e., academic and technical) remained limited (Honzard and Soyoof, 2020; Yüksel et al., 2020), and there is a need for further empirical research to understand both short- and long-term impacts of such interventions. Third, previous research indicated there is a considerable disciplinary variation in the way items from corpus-based word lists (such as AWL) are used in academic discourse (Hyland and Tse, 2007). As a result, teaching all items in a core academic wordlist for students in a particular field of study is not practical as many words in such lists are not relevant to their vocabulary learning needs. Fourth, with the expanding “centrality of English as a *lingua franca*” in all academic disciplines, there is a need for exploring new avenues for university students “to learn English in contexts that are meaningful to them” (Soyoof et al., 2022, p. 5). Accordingly, the present study used the results of a large corpus-based study of Psychology texts (Valizadeh and Xodabande, 2021) for selecting target academic words and connected findings from a corpus-based study of specialized texts to mobile-assisted vocabulary learning. The following research question was investigated:

Does using DFs on mobile devices result in enhanced learning outcome in teaching academic vocabulary?

## Materials and Methods

### Participants

The participants of the current study were 86 Iranian university students (49 females, 37 males) majoring in Psychology, recruited through purposive sampling. The following criteria were implemented in selecting the participants: nationality (i.e., Iranian), education level (i.e., Psychology major), and limited or no prior experience in living in an English-speaking country. The mean age of the participants was 22, and the majority were at the intermediate level in English based on (1) responses to the self-report proficiency measure, and (2) the results of the Cambridge Placement Test (Test Your English, 2022). The test is an online instrument with 25 multiple-choice format questions, which is used as a quick placement test for English language learners. It takes around 10 to 15 min to complete the test. At the time of the study, the participants were taking the course “English for Psychology students” as part of their 4-credit English for Specific Purposes (ESP) education. The course aimed to familiarize the students with reading disciplinary texts in Psychology. The participants were randomly assigned to three learning conditions. The experimental group 1 (*N* = 31) used digital flashcards on their mobile devices, the experimental group 2 (*N* = 30) used paper-based flashcards, and the control group (*N* = 25) was given a list of target vocabulary items with associated definitions. All participants in the experimental group 1 owned smartphone or tablet devices for installing and using vocabulary-learning applications. The study adhered to ethical considerations in educational research by obtaining informed consent from participants and ensuring the confidentiality of the collected data.

### Materials and Instruments

#### NAWL Builder Application

The study used New Academic Word List (NAWL; Browne et al., 2013) as a source for academic vocabulary in English. Accordingly, those participants in experimental group 1 installed the NAWL builder flashcards (EFL Technologies, 2017) to learn 361 academic words frequently used in Psychology (Valizadeh and Xodabande, 2021). This application is selected for this study for several reasons. First, it is freely accessible from the Google Play Store and Apple’s App Store for Android and iOS platforms. Second, using a built-in spaced repetition system (Kornell, 2009), the application facilitates the learning of vocabulary items in the NAWL list (Browne et al., 2013). Third, the NAWL builder employs a set of simple tools for learning vocabulary which makes it easy to use for language learners and keeps a detailed record of the users’ progress in vocabulary learning that can be emailed to the teacher. Moreover, the application uses simple English in the definitions given for academic vocabulary and provides part of speech information and North American pronunciation for the target words. In order to compare learning gains from different materials, the experimental group 2 was given ready-made paper flashcards for learning the same words that contained word form and related part of speech information on one side of the card, and simple definitions on the other side. The content of these word cards was similar to the cards in NAWL builder, except for the audio component of words. The control group was given a list of 361 academic words with their part of speech information and definitions.

#### Vocabulary Tests

In order to test the participants’ vocabulary knowledge before and following the treatment, two measures of receptive knowledge of academic vocabulary were employed. In this regard, New Academic Word List Test (NAWLT; Stoeckel and Bennett, 2020) was used as the first measure, which is a standard and validated diagnostic test of written receptive knowledge of vocabulary items in the NAWL. The NAWLT contains 40 items in multiple-choice format, and short sentences containing the target word in a natural but non-defining context are provided in the questions. The development of the NAWLT items was based on sound specifications, and the test in general shows high reliability (Cronbach’s alpha = 0.75). Moreover, two Vocabulary Knowledge Tests (VKT) each containing 60 multiple-choice items were developed to test the knowledge of 361 academic words frequently used in Psychology texts. For designing these tests, 120 items out of 361 words were selected randomly and assigned to two sets using the research randomizer website. The distractors in these tests were selected from simple definitions provided for NAWL items. The reliability of the developed test was acceptable (Cronbach’s alpha = 0.83), and its validity was examined in relation to the NAWLT in a pilot testing session on a similar sample (*N* = 20), and the test demonstrated acceptable concurrent validity (Frey, 2018) with an established instrument.

### Procedures and Data Analysis

At the start of the academic semester, the participants’ vocabulary knowledge was tested using the above-mentioned measures. This initial assessment of the vocabulary learning needs was followed by a one-hour training session for all participants on vocabulary learning strategies with focusing on digital flashcards and word lists. Then, the participants in the experimental group 2 and the control group were given ready-made flashcards and the word list, respectively. The participants in experimental group 1 installed the NAWL builder application and received instructions for selecting the 361 academic words that are relevant to Psychology. To this end, printed copies of the list of the frequently used NAWL items in Psychology (Valizadeh and Xodabande, 2021) which were sorted in alphabetical order were given to the participants, and they were asked to select the vocabulary items in the list^1^.

As part of their ESP course requirements, the participants in the two experimental groups were asked to spend at least 50 min every week (10–15 min per day) to study the target words (around 25 words per week) with the assigned materials over the course of an academic semester (i.e., 15 weeks). Academic vocabulary learning accounted for 30% of the overall course grade for the participants in the experimental groups and not for the control group. At the end of the academic semester, the participants’ vocabulary knowledge was tested again, to compare the learning outcomes across three learning conditions. The data obtained *via* vocabulary knowledge tests were analyzed using IBM SPSS (version 25) for both descriptive and inferential statistics. One-way between-groups ANOVA was performed to compare the scores on academic vocabulary tests on pre- and post-test.

## Results

Table 1 provides a summary of descriptive statistics for the results of the two tests, namely the NAWLT and VKT obtained by the participants on pre-test. As is shown in the table, the mean values calculated for both tests are consistent across the three groups. The total mean value for the scores was 11.73 (*SD* = 2.11) for the NAWLT, and the performances of the three groups were largely similar. With respect to the VKS, the total mean score for the participants was 20.28 (*SD* = 2.86).

**Table 1:** Descriptive statistics for pre-test results.

		*N*	Mean	Std. deviation	Std. error	95% confidence interval for mean	
						Lower bound	Upper bound
NAWLT	EXP 1	31	11.87	2.232	0.401	10.83	11.69
	EXP 2	30	11.93	2.033	0.371	11.17	12.69
	CON	25	11.32	2.076	0.415	10.46	12.18
	Total	86	11.73	2.111	0.228	11.28	12.19
VKT	EXP 1	31	20.23	2.997	0.538	19.13	21.33
	EXP 2	30	20.80	2.578	0.471	19.84	21.76
	CON	25	19.72	3.007	0.601	18.48	20.96
	Total	86	20.28	2.860	0.308	19.67	20.89

In order to see if the observed variation in the scores obtained by the three groups on the pre-test is statistically significant, a one-way between-groups ANOVA was conducted. Table 2 shows the results of Levene’s test for homogeneity of variances that investigates whether the observed variance in the scores is the same for the three groups. Since the test returned a non-significant results, the homogeneity assumption of variance has not been violated for the scores obtained on the pre-test.

**Table 2:** Test of homogeneity of variances for scores on pre-test.

		Levene’s statistic	df1	df2	Sig.
NAWLT	Based on mean	0.363	2	83	0.697
	Based on median	0.285	2	83	0.752
	Based on median and with adjusted df	0.285	2	80.090	0.753
	Based on trimmed mean	0.350	2	83	0.706
VKT	Based on mean	0.649	2	83	0.525
	Based on median	0.522	2	83	0.595
	Based on median and with adjusted df	0.522	2	77.808	0.595
	Based on trimmed mean	0.629	2	83	0.536

The results of the one-way between-groups analysis of variance (Table 3) revealed that the observed differences in the scores for the NAWLT, *F* (2, 83) = 0.67, *p* = 0.512, and the VKT, F (2, 83) = 0.98, *p* = 0.38, were not statistically significant. Accordingly, the results indicated that prior to the treatment, the three groups were similar in terms of their receptive knowledge of the 361 target academic words.

**Table 3:** One-way between-groups ANOVA for the scores on pre-test.

ANOVA						
		Sum of squares	df	Mean square	*F*	Sig.
NAWLT	Between groups	6.058	2	3.029	0.674	0.512
	Within groups	372.791	83	4.491		
	Total	378.849	85			
VKT	Between groups	16.043	2	8.021	0.980	0.380
	Within groups	679.259	83	8.184		
	Total	695.302	85			

The results of the descriptive statistics for the scores obtained on the post-test are summarized in Table 4. Unlike the participants’ performances on NAWLT and VKT on the pre-test, the post-test results show different learning outcomes for the three learning conditions. With respect to the NAWLT, the experimental group 1 obtained higher scores (*M* = 20.42, *SD* = 3.68), followed by the experimental group 2 (*M* = 16.20, *SD* = 3.15) and the control group (*M* = 13.12, *SD* = 3.00). As for the VKT, the post-test scores indicated a similar pattern, as the experimental group 1 obtained better scores (*M* = 41.00, *SD* = 4.75). The participants in the experimental group 2 had higher scores (*M* = 34.73, *SD* = 5.89) compared to the control group (*M* = 30.92, *SD* = 6.99).

**Table 4:** Descriptive statistics for post-test results.

		*N*	Mean	Std. deviation	Std. error	95% confidence interval for mean	
						Lower bound	Upper bound
NAWLT	EXP 1	31	20.42	3.686	0.662	19.07	21.77
	EXP 2	30	16.20	3.156	0.576	15.02	17.38
	CON	25	13.12	3.004	0.601	11.88	14.36
	Total	86	16.83	4.430	0.478	15.88	17.78
VKT	EXP 1	31	41.00	4.754	0.854	39.26	42.74
	EXP 2	30	34.73	5.889	1.075	32.53	36.93
	CON	25	30.92	6.474	1.295	28.25	33.59
	Total	86	35.88	6.993	0.754	34.38	37.38

In order to proceed with analyzing the data for inferential statistics, Levene’s test for homogeneity of variances was conducted prior to ANOVA, and the results (Table 5) indicated that the assumption of homogeneity of variance has not been violated for the scores obtained on post-test. Additionally, the results of the one-way between-groups analysis of variance (Table 6) revealed that the observed differences in the scores for the NAWLT, F (2, 83) = 34.33, *p* < 0.001; eta squared = 0.45), and the VKT, F (2, 83) = 22.63, p < 0.001; eta squared = 0.35, were statistically significant. Accordingly, the results indicated that after the treatment, the three groups were different in terms of their receptive knowledge of the 361 target academic words. The effect size of the observed differences for both measures was very large based on the criteria proposed by Cohen (1988).

**Table 5:** Test of homogeneity of variances for scores on post-test.

		Levene’s statistic	df1	df2	Sig.
NAWLT	Based on mean	1.636	2	83	0.201
	Based on median	1.333	2	83	0.269
	Based on median and with adjusted df	1.333	2	79.169	0.270
	Based on trimmed mean	1.633	2	83	0.202
VKT	Based on mean	1.956	2	83	0.148
	Based on median	1.547	2	83	0.219
	Based on median and with adjusted df	1.547	2	76.836	0.220
	Based on trimmed mean	1.923	2	83	0.153

**Table 6:** One-way between-groups ANOVA for the scores on post-test.

ANOVA						
		Sum of squares	df	Mean square	*F*	Sig.
NAWLT	Between groups	755.395	2	377.698	34.337	0.000
	Within groups	912.988	83	11.000		
	Total	1668.384	85			
VKT	Between groups	1467.131	2	733.565	22.637	0.000
	Within groups	2689.707	83	32.406		
	Total	4156.837	85			

Finally, in order to compare and contrast the scores obtained by the three groups on the post-test, a series of pairwise comparisons were conducted (Table 7). The results revealed that the experimental group 1 that used DFs for learning academic vocabulary outperformed the experimental group 2 (mean differences: NAWLT = 4.22, VKT = 6.27, *p* < 0.001) and the control group (mean differences: NAWLT = 7.29, VKT = 10.08, *p* < 0.001). Moreover, the participants in experimental group 2 that used paper flashcards for vocabulary learning outperformed the control group (mean differences: NAWLT = 3.08, VKT = 3.81, p < 0.001).

**Table 7:** Multiple comparisons.

Tukey’s HSD							
Dependent variable	(I) Group	(J) Group	Mean difference (I-J)	Std. error	Sig.	95% confidence interval	
						Lower bound	Upper bound
NAWLT	EXP 1	EXP 2	4.219^*^	0.849	0.000	2.19	6.25
		CON	7.299^*^	0.892	0.000	5.17	9.43
	EXP 2	EXP 1	−4.219^*^	0.849	0.000	−6.25	−2.19
		CON	3.080^*^	0.898	0.003	0.94	5.22
	CON	EXP 1	−7.299^*^	0.892	0.000	−9.43	−5.17
		EXP 2	−3.080^*^	0.898	0.003	−5.22	−0.94
VKT2	EXP 1	EXP 2	6.267^*^	1.458	0.000	2.79	9.75
		CON	10.080^*^	1.530	0.000	6.43	13.73
	EXP 2	EXP 1	−6.267^*^	1.458	0.000	−9.75	−2.79
		CON	3.813^*^	1.542	0.040	0.13	7.49
	CON	EXP 1	−10.080^*^	1.530	0.000	−13.73	−6.43
		EXP 2	−3.813^*^	1.542	0.040	−7.49	−0.13

* The mean difference is significant at the 0.05 level.

## Discussion

The present study investigated the impacts of using DFs on mobile devices for learning academic vocabulary by university students and compared the learning outcomes to traditional materials including paper flashcards and word lists. The results indicated that mobile-assisted vocabulary learning using digital flashcards with built-in spaced repetition technology improved participants’ academic vocabulary knowledge significantly from pre-test to the post-test, and that the participants in the experimental group outperformed the other groups on both measures of academic vocabulary knowledge. These findings are congruent with earlier studies that reported positive learning outcomes for mobile-assisted vocabulary learning (Dizon, 2016; Ashcroft et al., 2018; Kohnke et al., 2019, 2020; Xodabande and Atai, 2020; Yüksel et al., 2020). A close examination of the results obtained on pre- and post-tests on VKT (Tables 1, 4) revealed that prior to the treatment, the participants of the study were familiar with about 33% of the 361 academic words (around 120 items) that are frequently used in their field of study. However, after the semester-long treatment/instruction, the participants in the experimental group 1 learned around 126 more items and their test results pointed to achieving around 68% mastery over the target items (35% improvement). Additionally, the participants in experimental group 2 learned around 56% of the target items (23% improvement), and those in the control group learned 51% of the items (18% improvement). Accordingly, although the interventions were not effective in teaching all 361 words, considerable improvements in the vocabulary knowledge of the participants in the experimental learning conditions point to the effectiveness of explicit focus on vocabulary learning in general and the relative advantage of mobile-assisted learning in the target items in particular. It has been argued that the integration of digital technologies into language education positively impacts learners’ motivation (Stockwell, 2013), and the motivational dimension of mobile-assisted vocabulary learning might be considered the key factor explaining the significant learning outcomes in the experimental group 1. Additionally, studies indicated learning vocabulary items alongside multimedia features such as pictures and audio pronunciation simultaneously improve learning outcomes (Rasti-Behbahani and Shahbazi, 2020), which partly explains better learning outcomes for digital flashcards observed in this study. Another factor that further contributed to overall improvements in the test scores might be the integration of the academic vocabulary learning component into the ongoing ESP course for the participants, which resulted in increased engagement with materials and spending more time and effort for learning target words.

Moreover, the treatment in the form of mobile-assisted vocabulary learning lasted for a semester, and the findings of the study supported the long-term effectiveness of mobile devices and DFs in teaching academic vocabulary to university students. This is also congruent with the limited findings in the literature that reported long-term positive outcomes for mobile-assisted academic vocabulary learning (Lin and Lin, 2019). As the majority of previous studies on mobile-assisted language learning was conducted in short time periods (Chwo et al., 2018; Lin and Lin, 2019), these results are significant as they add to the growing body of knowledge in mobile-assisted learning of general and specialized vocabulary. The observed long-term effectiveness of mobile-assisted vocabulary learning might be attributed to a number of factors. First, since the target words were selected from a corpus-based study of specialized texts in Psychology, the vocabulary items were highly relevant to the participant’s field of study, which might have resulted in their increased motivation and learning effort. Second, as the NAWL builder app has a built-in spaced repetition feature, the participants learned and practiced academic vocabulary systematically and efficiently. Additionally, the availability of mobile devices and learning materials for the participants facilitated anytime and anywhere learning experience (Lin and Lin, 2019) which promoted learning outcomes over time.

Moreover, given the relatively large number of items in core academic wordlists, focusing on those words that are more frequent in a given field of study brings better learning outcomes for some reasons. First, reducing the number of target vocabulary items makes the list more manageable for students to study the vocabulary items with DFs in one or two semesters. Second, as the items are highly relevant to their discipline and professional identity, university students might be more motivated to invest time and effort in learning discipline-related/specific academic words. Third, as vocabulary instruction receives insufficient attention in language classes (Webb and Nation, 2017), such a fine-tuned approach allows instructors and students to use valuable classroom and self-study time for focusing on the most important academic vocabulary items. Finally, although the findings of the study pointed to the long-term effectiveness of the DFs in academic vocabulary learning, obtaining slightly lower (but statistically significant) scores on the delayed post-test by the participants shows that without reinforcing the developing knowledge of vocabulary items, the learning gains might be lost over time. As a result, there is a need for systematic review and passive or active use of learned items through academic reading and writing.

## Conclusion and Implications

The current study pursued two main goals: (a) exploring the effect of using DFs on learning academic vocabulary among a group of Psychology major university students and (b) comparing the learning outcomes from DFs to traditional materials. Designed as a semester-long experimental study, the results indicated that using DFs inherently could enhance students’ engagement with learning their discipline-specific vocabulary items during the intervention. The findings also indicated that participants using DFs on their mobile devices outperformed the participants using paper flashcards and word lists in vocabulary learning. Moreover, the long-term impact of the interventions could be noteworthy with regard to sustaining students’ vocabulary retention rate at a higher level compared to the pre-treatment levels. The findings from the study generally add to the growing literature on mobile-assisted language learning by providing empirical support on the effectiveness of mobile-assisted vocabulary learning among students who have limited and discipline-specific exposure to English and minimal opportunity to utilize the gained knowledge outside the given academic context. In other words, these devices and technologies would act as facilitators and scaffolds in directing students’ learning and enhancing their autonomy in taking control of their learning and hence practicing their agency in such a process.

The current study has implications for teaching academic vocabulary to university students. As English has established itself as the *lingua franca* for academic publication in international journals, university students are increasingly required to read and publish in English. In this regard, English has become much more instrumental in shaping their academic identity that also facilitates their access to the pertinent literature in their discipline (Paquot, 2010). Nevertheless, studies in academic writing indicated that English users with non-English linguistic backgrounds in particular face consequential linguistic impediments in such undertakings (Flowerdew, 2015), and inadequate vocabulary knowledge is one of the most important factors that add to their disadvantage in academic publishing (Bazerman et al., 2012). Given the significant role of academic vocabulary in university studies, mastery over core academic words benefits university students and EAP programs in many ways. The findings of the current study indicated that using DFs and mobile applications provided the participants with the affordances to learn a considerable number of academic words frequently used in their disciplines. Accordingly, instructors and materials developers might consider adding them to EAP programs. Additionally, university students can use well-designed DF applications for developing their academic vocabulary knowledge in self-directed learning.

The study, however, has some limitations. First, the research was conducted with two relatively small experimental groups each containing around 30 participants and a control group with 25 individuals. This should be accounted for in generalizing the findings, as the small sample size in each group might have resulted in biased results. Second, given that the study lasted for a semester, controlling the students’ possible exposure and contact with other language learning materials was not possible. In this regard, although they had no considerable exposure to other materials for learning academic vocabulary beyond the classroom, some learning might have resulted from other resources (Xodabande, 2018). Additionally, as vocabulary knowledge has different aspects and entails both receptive and productive uses of words (Nation, 2013), the current study was concerned with developing the participants’ receptive knowledge of academic words only. This focus on receptive knowledge was in line with the participants’ vocabulary learning needs (i.e., to read specialized and academic texts); however, it should be acknowledged that any intervention to develop university students’ productive vocabulary knowledge is much more important (Soyoof et al., 2022). Despite these limitations, the study was conducted in a longer time span with different measurements administered prior to and after the treatment, and the findings provided additional empirical evidence supporting the affordances of DFs and mobile devices for academic vocabulary learning in the EFL context. Future research might consider investigating the impacts of DFs not only on receptive knowledge of academic words but also on their productive use in speaking and writing.

## Data Availability Statement

The datasets used and/or analyzed during the current study are available from the corresponding author on reasonable request.

## Ethics Statement

Ethical review and approval was not required for the study on human participants in accordance with the local legislation and institutional requirements. The patients/participants provided their written informed consent to participate in this study.

## Author Contributions

All authors listed have made a substantial, direct, and intellectual contribution to the work and approved it for publication.

## Conflict of Interest

The authors declare that the research was conducted in the absence of any commercial or financial relationships that could be construed as a potential conflict of interest.

## Publisher’s Note

All claims expressed in this article are solely those of the authors and do not necessarily represent those of their affiliated organizations, or those of the publisher, the editors and the reviewers. Any product that may be evaluated in this article, or claim that may be made by its manufacturer, is not guaranteed or endorsed by the publisher.

## Footnotes

^1^ The NAWL software can be accessed at: https://apps.apple.com/us/app/nawl-builder-multilingual/id1097693935.
